# Supplementary material for: Content-rich biological network constructed by mining PubMed abstracts
Source: BMC Bioinformatics. 2004 Oct 8;5:147. doi: 10.1186/1471-2105-5-147 (PMC528731; doi:10.1186/1471-2105-5-147)
Supplement: Additional File 5 — The original Chilibot query results of the term "long-term potentiation (LTP)" and 22 other terms, limiting the latest references analyzed to the years 1990, 1995, 2000, and 2004. [file 1471-2105-5-147-S5.bz2 › chilibotAdditionalFile5/ltp2004/html/TRKA_SYNAPTOPHYSIN.html]

 


 **TRKA** and **SYNAPTOPHYSIN** 
  
Found 6 abstracts in PubMed,  **6 abstracts were retrieved and analyzed**.  


---

 Search Google  |
 PDF files only 
|  EDU domain only 

---

**Interactive relationship** (e.g. stimulation, inhibition, etc)

- The concentrations of norepinephrine, the major sympathetic neurotransmitter, and growth cone located  **synaptophysin** , a neurosecretory granule protein, were increased in NGF treated SH SY5Y  **trkA**  but not in NT 3 treated SH SY5Y trkC cells.  Ref: 11205744 Cell Growth Differ, 2001

- :-)

  **Parallel relationship** (e.g. studied together, co-existance, homology, etc.)

  - In addition to routine histological examinations, immunohistochemistry was used to evaluate the expression of neurotrophin receptors  **TrkA** , TrkB and of neuronal differentiation markers such as neuron specific enolase, neurofilament,  **synaptophysin** , and chromogranin A.  Ref: 9625303 Clin NeuropatholClin Neuropathol, 2001
